# Supplementary material for: ASFV infection induces macrophage necroptosis and releases proinflammatory cytokine by ZBP1-RIPK3-MLKL necrosome activation
Source: Front Microbiol. 2024 Jun 17;15:1419615. doi: 10.3389/fmicb.2024.1419615 (PMC11215146; doi:10.3389/fmicb.2024.1419615)
Supplement: Supplementary file 1 [file Image_1.pdf]

# *Supplementary Material*

## Supplementary Figures

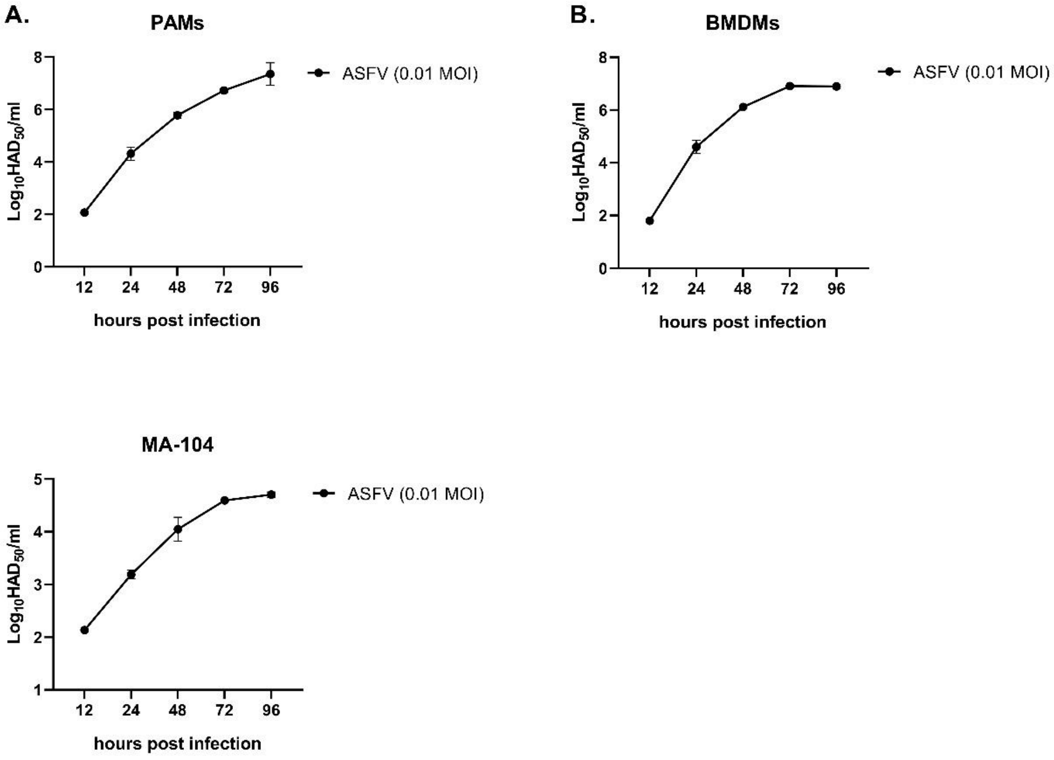


**Supplementary Figure 1.** The proliferation kinetics of ASFV in diffierent cells. PAMs (A), BMDMs (B) and MA-104 cells (C) were infected with ASFV (MOI = 0.01), and the viral titers at 12, 24, 48, 72, and 96 hpi were determined using the HAD50 method. Data are presented as mean ± SD of three independent experiments. Heatmap of cytokine gene expression profiles after FIPV infection.

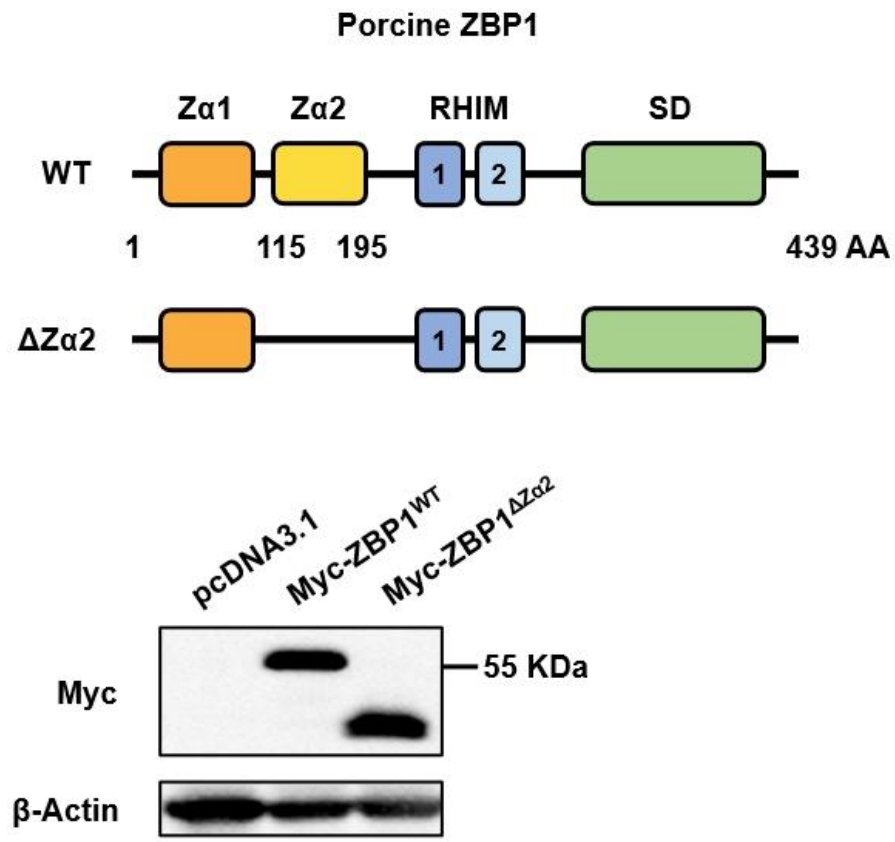


**Supplementary Figure 2.** The construction and expression of Myc-tagged porcine ZBP1WT and ZBP1ΔZα2 plasmids.
